# Association Between Perioperative Glycemic Control Strategy and Mortality in Patients With Diabetes Undergoing Cardiac Surgery: A Systematic Review and Meta-Analysis

**DOI:** 10.3389/fendo.2020.513073

**Published:** 2020-12-17

**Authors:** Xinye Jin, Jinjing Wang, Yanfang Ma, Xueqiong Li, Ping An, Jie Wang, Wenfeng Mao, Yiming Mu, Yaolong Chen, Kang Chen

**Affiliations:** ^1^ Department of Endocrinology, Chinese PLA General Hospital, Beijing, China; ^2^ Department of Endocrinology and Nephrology, Hainan Hospital of Chinese PLA General Hospital, Sanya, China; ^3^ Hainan Academician Team Innovation Center, Sanya, China; ^4^ Department of Endocrinology, Fifth Medical Center of Chinese PLA General Hospital, Beijing, China; ^5^ Evidence-Based Medicine Center, School of Basic Medical Sciences, Lanzhou University, Lanzhou, China; ^6^ WHO Collaborating Center for Guideline Implementation and Knowledge Translation, Lanzhou, China; ^7^ Chinese GRADE Center, Lanzhou, China; ^8^ Department of Gerontology, First Affiliated Hospital of Kunming Medical University, Kunming, China; ^9^ School of Medicine, Nankai University, Tianjin, China

**Keywords:** meta-analysis, systematic review, cardiac surgery, perioperative glycemic control, diabetes

## Abstract

**Objective:**

To analyze association between different perioperative glycemic control strategies and postoperative outcomes in patients with diabetes undergoing cardiac surgery.

**Methods:**

MEDLINE, Cochrane Library, Web of Science, EMBASE, Wanfang Data, China National Knowledge Infrastructure (CNKI) and China Biology Medicine (CBM) databases were searched from inception to January 31, 2019. Two researchers independently screened the literature, extracted data, and evaluated the risk of bias of included studies, and consensus was reached by discussion with a third researcher.

**Results:**

Six RCTs were included in the meta-analysis. We analyzed the effect of liberal (>180 mg/dl or 10.0 mmol/L), moderate (140–180 mg/dl or 7.8–10.0 mmol/L) and strict (<140 mg/dl or 7.8 mmol/L) glycemic control strategies in patients with diabetes undergoing cardiac surgery. The pooled results showed that strict glycemic control strategy was associated with a significant reduction in the risk of atrial fibrillation [OR = 0.48, 95%CI (0.32, 0.72), *P* < 0.001] and sternal wound infection [OR = 0.28, 95%CI (0.14, 0.54), *P* < 0.001], while there was no significant differences in postoperative mortality, stroke, and hypoglycemic episodes when compared with moderate control. In addition, there is no significant difference between moderate and liberal glycemic control strategies in postoperative mortality. However, moderate control was beneficial in reducing atrial fibrillation [OR = 0.28, 95%CI (0.13, 0.60), *P* = 0.001] compared with the liberal glycemic control strategy.

**Conclusions:**

This meta-analysis showed when compared with moderate glycemic control strategy in patients with diabetes undergoing cardiac surgery, maintained strict glycemic control was associated with lower risk of atrial fibrillation and sternal wound infection. No benefit was found with liberal glycemic control strategy, so it could be a poor glycemic control strategy.

## Introduction

In recent years, the prevalence of diabetes has increased dramatically worldwide. According to the statistics released by the International Diabetes Federation (IDF), there were 425 million patients with diabetes worldwide up to 2017 (1). In China, the overall prevalence of diabetes in adults is 11.6%, and the total number of patients ranks first in the world (2). At the same time, as the number of people with diabetes increased, so did the proportion of diabetics who need surgery. Furthermore, diabetics are more likely to undergo certain surgeries than non-diabetics, and they have high morbidity and mortality rates when seriously deteriorated or ill (3). Among all patients undergoing surgery, diabetes accounts for 5%, and this proportion can reach up to 10% in elderly patients (4).

Perioperative hyperglycemia has been associated with increased diabetic keto-acidosis (5–7). Severe hyperglycemia could lead to dehydration and hypokalemia caused by osmotic diuresis and increase the risk of mechanical ventilation dependence, fibrillation, wound infection, and death (8, 9). Inversely, severe hypoglycemia could lead to cognitive dysfunction and brain death (1, 2, 10). However, the perioperative glycemic control standards for cardiac surgery are still not uniform. The randomized controlled trial (RCT) results of Berghe et al. (11) showed that the glucose levels of adults admitted to surgical intensive care unit who received mechanical ventilation were strictly controlled at 4.4–6.1 mmol/L, and the hospital mortality rate decreased by 34%. Yates et al. (12) conducted RCT in children undergoing cardiac surgery, and the results showed that postoperative hyperglycemia increased the incidence of complications and mortality, so they advocate strict perioperative glycemic control. On the contrary, the results of Gandhi’s study showed intensive insulin therapy during cardiac surgery increased the incidence of death and stroke (13). Diabetics are more prone to hyperglycemia during perioperative period, so it is essential for doctors to control glycemia in diabetes patients. However, the optimal perioperative glycemic targets for patients with diabetes are still unclear. The current international diabetes guidelines for inpatient glycemic targets are largely based on critically ill patients who may or may not have undergo surgery (14, 15), and the existing trials for surgical patients included both diabetics and non-diabetics (14, 16). However, the glycemic control strategy is different between diabetics and non-diabetics during the perioperative period (16). Therefore, it is necessary to develop perioperative glycemic control strategies for patients with diabetes (17, 18).

Different types of surgeries were associated with different clinical outcomes in patients with diabetes. Several trials have studied glycemic control strategies for different types of surgery (19–24). In this study, our aim is to analyze the relationship between the different perioperative glycemic control strategies and postoperative outcomes in patients with diabetes undergoing cardiac surgery.

## Materials and Methods

### Perioperative Glycemic Targets

In view of international guidelines, there is no recommendation for perioperative glycemic control in patients with type 1 and type 2 diabetes undergoing cardiac surgery (15). Therefore, we defined the intensity of perioperative glycemic control based on the glycemic targets which set by studies included in this systematic review (19–24). According to the relevant domestic and international guidelines for diabetes (25, 26), we applied 140 mg/dl (7.8 mmol/L) and 180 mg/dl (10.0 mmol/L) as the cut-off points of perioperative glycemic control targets. Hence, glycemic control was considered as strict when its target is less than 140 mg/dl (7.8 mmol/L), moderate when it ranges within 140–180 mg/dl (7.8–10.0 mmol/L), and liberal when it’s more than 180 mg/dl (10.0 mmol/L). Given the spectrum of the reported glycemic targets, studies were classified into strict, moderate, or liberal glycemic control strategy.

### Search Strategy

MEDLINE, Cochrane Library, Web of Science, EMBASE were searched for studies examining the effects of different perioperative glycemic control strategies in patients with diabetes undergoing cardiac surgery. In addition, Chinese databases, including Wanfang Data, China National Knowledge Infrastructure (CNKI) and Chinese Biology Medicine (CBM) were also searched. The search was not limited by date of publication, but limited to studies published in English and Chinese, and pertaining to human subjects from inception to January 31, 2019. The search was performed using the medical subject heading (MeSH) terms and text words, such as “diabetes mellitus” AND “perioperative” AND “heart surgery” (see **Supplementary 1** for more details).

### Selection Criteria

The identified titles and abstracts were independently reviewed by two researchers (XJ and JW). Inclusion criteria for identified studies were: (1) Patients diagnosed with diabetes, including type 1 and type 2 diabetes, (2) adult population, (3) undergoing heart surgery, (4) presence of perioperative glycemic measurements, (5) at least two different perioperative glycemic control targets for comparison within the same evaluation period, (6) randomized controlled trial (RCT) or cohort studies, historic controls (9, 27, 28). Newcastle-Ottawa Scale (NOS) score <6, conference abstracts, emergency heart surgeries, and duplicates were excluded.

### Data Extraction

Two researchers (XJ and JW) independently extracted the following information from the selected studies: study design, sample size, baseline patient characteristics, mean plasma glucose (PG) achieved in each group, glycemic targets, timing of intervention (intraoperative or postoperative), mortality data, rates of atrial fibrillation, stroke, hypoglycemia, and infection. Disagreements over study selection were resolved through discussion with a third researcher (YM). If data from the full text were not reported clearly, we tried to contact the corresponding author to obtain the original/relevant data. If the author did not respond within one month, we excluded the study.

### Study Outcomes

The primary outcome in this study was mortality. The secondary outcomes were postoperative incidence of stroke, atrial fibrillation, wound infection, and hypoglycemia episodes.

### Risk of Bias Assessment

Two researchers (XJ and JW) independently evaluated the risk of bias for the included RCTs based on the Cochrane handbook (29), and risk of bias of cohort studies was assessed using the NOS checklist (30). If the NOS score <6, which indicated the study had serious bias, we excluded the study from the meta-analysis. In order to make sure that each reviewer understood the items clearly, we conducted a pilot study before formal assessment. Disagreements were resolved by discussion with a third researcher (YM).

### Data Synthesis

The effect sizes expressed as odds ratios (ORs) and their 95% confidence intervals (CIs) were calculated by using the random or fixed effects model (31). Heterogeneity was estimated by using *I* ^2^ statistics (describes the percentage of variation across studies due to heterogeneity rather than chance) and tested by using the corresponding Chi-square statistics. Significant heterogeneity was present when *I* ^2^>50% or a *p* value of <0.10 in the chi-square statistic. In order to determine whether any single trial substantially influences results, after systematically eliminating one trial each time, the sensitivity analysis was conducted for the primary outcome by recalculating the pooled estimate. When the number of inclusion indicators is greater than or equal to 10, the publication bias was evaluated by making a funnel plot. The statistical analysis was performed by using Cochrane Collaboration’s Review Manager 5.3 software.

### Quality of Evidence Assessment

The quality of evidence for each outcome was assessed and graded by using the GRADE (Grading of Recommendations Assessment, Development and Evaluation) system (32–38) recommended by the Cochrane Collaboration. The judgments of quality for specific outcomes were based on five main areas: study design and execution limitations, inconsistency, indirectness, imprecision of results, and publication bias across all studies. The overall quality of evidence for each outcome was combined all above areas’ assessments and was graded as very low, low, moderate, or high for recommendations.

## Results

### Search Results

**Figure 1:**
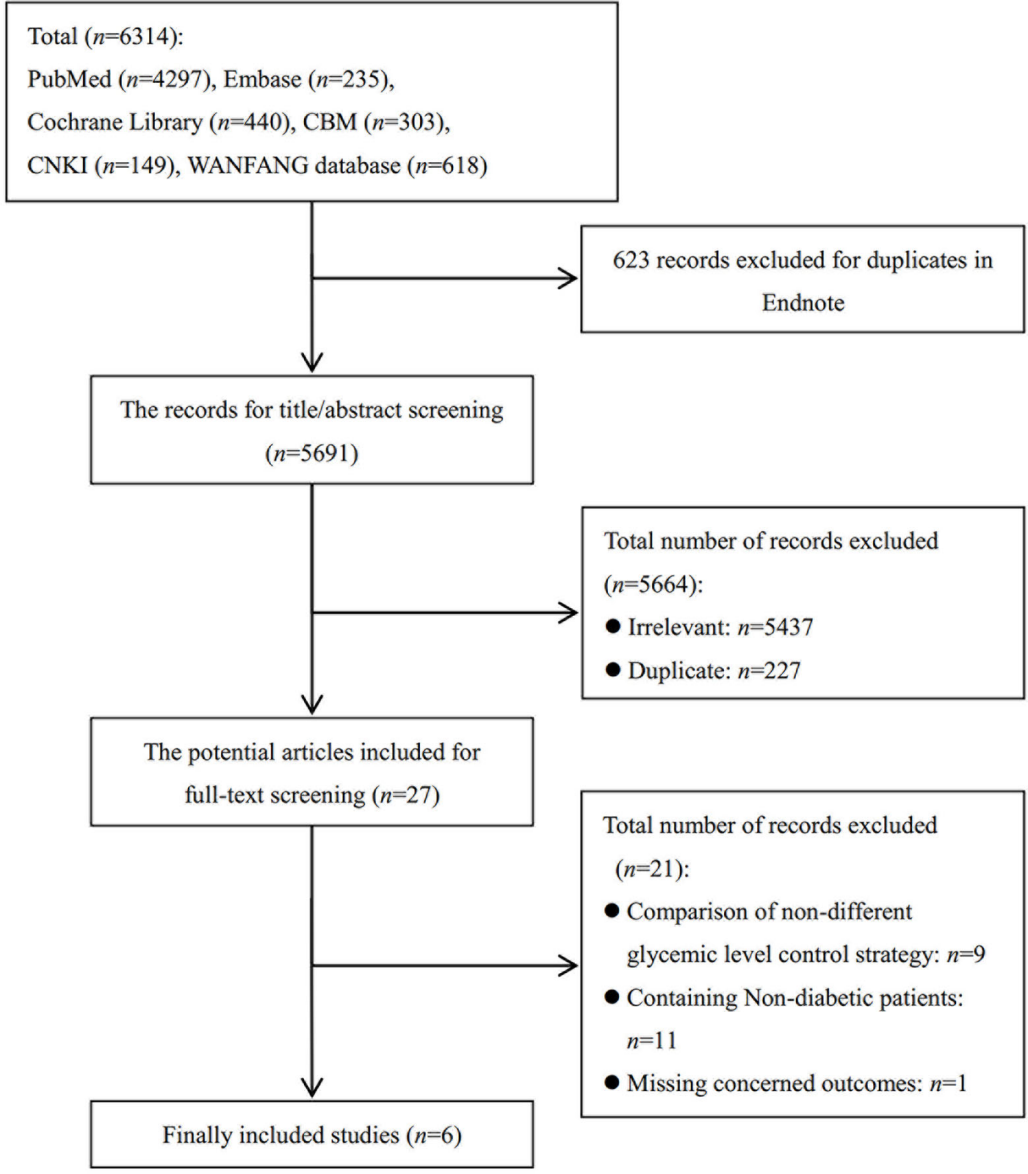
Flow diagram illustrating the process of identifying articles for selection.

The literature search identified 6,314 records (**Figure 1**). After 623 duplicates were removed with Endnote, 5,691 records’ titles and abstracts and full text were reviewed, and 5,664 were excluded. The remaining 27 studies underwent a full text review with 21 studies excluded due to inclusion of non-diabetics, non-concerned comparison, or lack of concerned outcomes. Finally, six RCTs (20–22, 39–41) were finally included in this meta-analysis. References (*n* = 21) excluded after reading full-text are provided in **Supplementary 2**.

### Characteristics of the Selected Studies

Six RCTs (20–22, 39–41) all included patients with diagnosed diabetes, but they did not specify their diagnostic criteria. Five studies (20–22, 40, 41) only reported that their patients were diabetics, and one study (39) clearly reported that they included both type 1 and type 2 diabetic patients. All studies were published between 2004 and 2016; the sample sizes ranged from 75 to 200. The mean age of patients was from 46 to 65. In terms of glucose evaluation, two studies (21, 22) directly reported it was measured in serum, three studies (20, 40, 41) were measured in blood, and one study (39) indicated it was venous blood samples. A differential in the timing of intervention was noted among the included studies, with five studies started glycemic control preoperatively (20–22, 40, 41), and one study started glycemic control intra-operatively (39). However, all the timing of glucose evaluation was at pre-operation and post-operation. In addition, the glycemic targets varied between trials. There were five studies compared strict and moderate glycemic control strategies (20, 21, 39–41), whereas the other one study compared moderate and liberal glycemic control strategies (22). All studies used insulin to control PG. Three studies reported 30-days mortality and two studies (20, 41) reported mortality measured in-hospital or in-ICU (Intensive Care Unit). Characteristic information of including studies is shown in **Table 1**.

**Table 1:** Characteristics of the included RCTs.

Study ID	Setting	Surgery type	Timing of intervention	Groups	Glucose-lowering treatment	N	Mean age	%Male	Preoperative mean PG (mg/dl)	PerioperativePG target (mg/dl)	Postoperative mean PG (mg/dl)
**Strict (target <140 mg/dl or 7.8 mmol/L) *vs.* Moderate (target 140180 mg/dl or 7.810.0 mmol/L)**											
Kirdemir et al. (20)	Turkey	CABG	Preoperative	Strict	continuous insulin infusion	100	58	59	178.6	100–150	172
				Moderate	Intermittent Subcutaneous Insulin	100	57	65	189.4	<200	195
Lazar et al. (21)	USA	CABG	Preoperative	Strict	continuous insulin infusion	40	63	80	/	90–120	103
				Moderate	continuous insulin infusion	42	65	61.9	/	120–180	135
Asida et al. (39)	Egypt	cardiac surgery	Intraoperative	Strict	continuous insulin infusion	50	46	60	147	80–110	106
				Moderate	conditional infusion of rapidly acting insulin	50	49	66.67	155	110–180	155
Wahby et al. (40)	Egypt	CABG	Preoperative	Strict	continuous insulin infusion	67	54.99	73.1	164.06	110–149	/
				Moderate	conventional moderate glycemic control	68	56.4	67.6	166.9	150–180	/
Zadeh and Nour (41)	Iran	cardiac surgery	Preoperative	Strict	infusion of regular insulin	38	56.4	44.74	190.68	100–120	/
				Moderate	conditional administer regular insulin	37	58.18	35.14	192.41	≤200	/
**Moderate (target 140**–**180 mg/dl or 7.8**–**10.0 mmol/L) *vs.* Liberal (target > 180 mg/dl or 10.0 mmol/L)**											
Lazar et al. (22)	USA	CABG	Preoperative	Moderate	modified glucose-insulin-potassium infusion	72	63.7	58.3	180.4	125–200	134.3
				Liberal	administer subcutaneous insulin	69	63.5	66.7	179.0	<250	266.8

RCT, Randomized Controlled Trial; CABG, Coronary Artery Bypass Graft.

### Risk of Bias of the Selected Studies

The results of the quality assessment for the six selected RCTs are presented in **Table 2**. Three studies (21, 22, 41) did not report the method of randomization. Five studies (20–22, 39, 40) did not report allocation concealment, and one study was an open-labeled trial (41). All six studies (20–22, 39–41) did not report blinding of participants and personnel and blinding of outcome assessment. Also, all of six studies (20–22, 39–41) had a low risk for incomplete outcome data and selective reporting.

**Table 2:** Risk of bias of the included RCTs.

Study ID	Random sequence generation	Allocation concealment	Blinding of participants and personnel	Blinding of outcome assessment	Incomplete outcome data	Selective reporting	Other bias
Lazar et al. (22)	Unclear	Unclear	High risk	Unclear	Low risk	Low risk	Unclear
Kirdemir et al. (20)	Low risk	Unclear	High risk	Unclear	Low risk	Low risk	Unclear
Lazar et al. (21)	Unclear	Unclear	High risk	Unclear	Low risk	Low risk	Unclear
Asida et al. (39)	Low risk	Unclear	High risk	Unclear	Low risk	Low risk	Unclear
Zadeh and Nour (41)	Unclear	High risk	High risk	Unclear	Low risk	Low risk	Unclear
Wahby et al. (40)	Low risk	Unclear	High risk	Unclear	Low risk	Low risk	Unclear

### Strict *vs* Moderate Glycemic Control Strategy

#### Mortality

**Figure 2:**
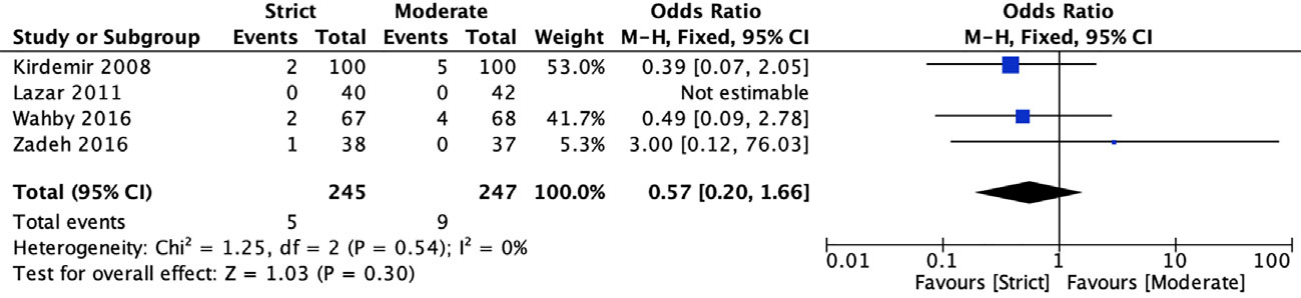
Forest plot for postoperative mortality between perioperative strict and moderate glycemic control strategy.

Four RCTs (20, 22, 40, 41) reported mortality for strict and moderate glycemic control strategy. The pooled results indicated there was no significant difference in the incidence of mortality [2.04 *vs* 3.64%, OR = 0.57, 95%CI (0.20, 1.66), *P* = 0.30] between strict and moderate glycemic control strategy, and no heterogeneity was found in the pooled estimates (*I* ^2^ = 0%, *P* = 0.54) (**Figure 2**).

#### Stroke

**Figure 3:**
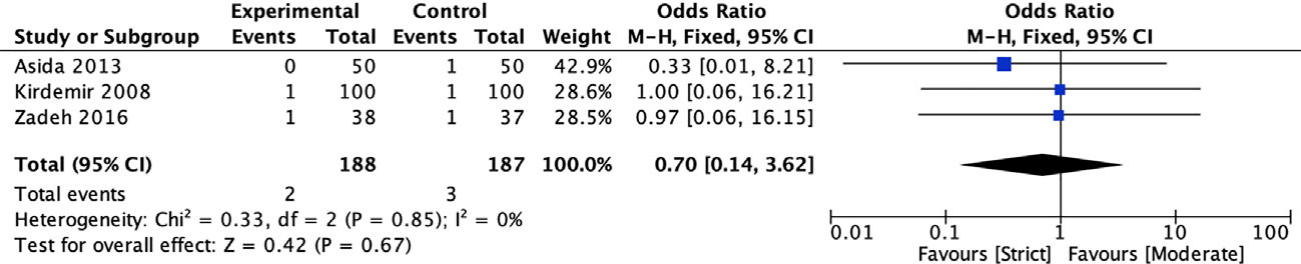
Forest plot for postoperative stroke between perioperative strict and moderate glycemic control strategy.

Three RCTs (20, 39, 41) compared the incidence of stroke for strict and moderate glycemic control strategy. The pooled results suggested there was no significant reduction in the incidence of stroke [1.06 *vs* 1.60%, OR = 0.70, 95%CI (0.14, 3.62), *P* = 0.67] between strict and moderate glycemic control strategy and no heterogeneity in the pooled estimates (*I* ^2^ = 0%, *P* = 0.85) (**Figure 3**).

#### Atrial fibrillation

**Figure 4:**
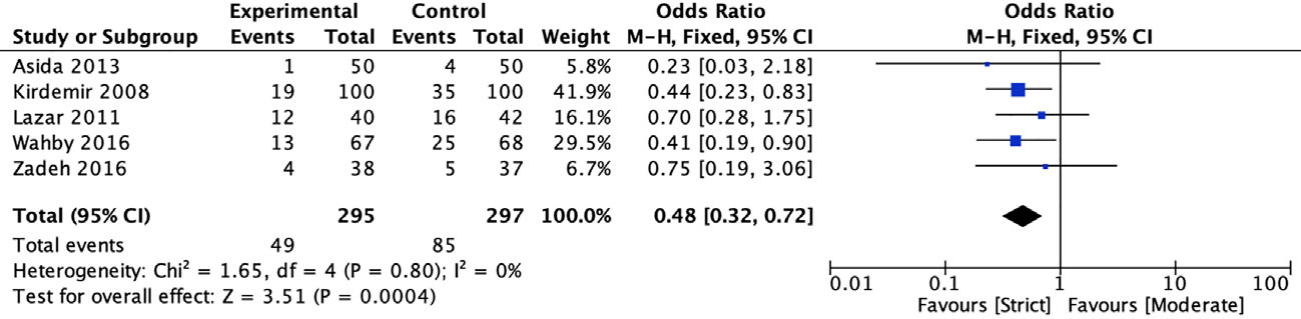
Forest plot for postoperative atrial fibrillation between perioperative strict and moderate glycemic control strategy.

Five studies (20, 22, 39–41) reported the incidence of atrial fibrillation between strict and moderate glycemic control strategy. The pooled estimates indicated that strict glycemic control strategy was associated with a significant reduction in the risk of atrial fibrillation *versus* moderate glycemic control strategy [16.61 *vs* 28.62%, OR = 0.48, 95%CI (0.32, 0.72), *P* < 0.001], and there was no heterogeneity in the pooled estimates (*I* ^2^ = 0%, *P* = 0.80) (**Figure 4**).

#### Sternal Wound infection

**Figure 5:**
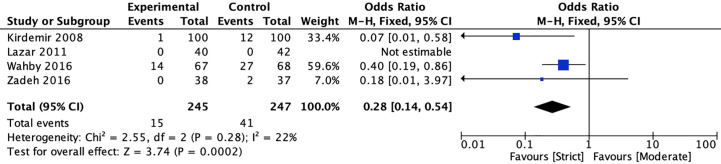
Forest plot for postoperative sternal wound infection between perioperative strict and moderate glycemic control strategy.

In terms of wound infection, four studies (20, 22, 40, 41) reported the incidence of sternal wound infection, and one study reported the incidence of leg wound infection between strict and moderate glycemic control strategy. The pooled estimates showed that strict glycemic control strategy was associated with a significant reduction in sternal wound infection versus moderate glycemic control strategy [6.12 *vs* 16.60%, OR = 0.28, 95%CI (0.14, 0.54), *P* < 0.001], and no significant heterogeneity was found in the pooled estimates (*I* ^2^ = 22%, *P* = 0.28) (**Figure 5**).

#### Hypoglycemic Episodes

**Figure 6:**
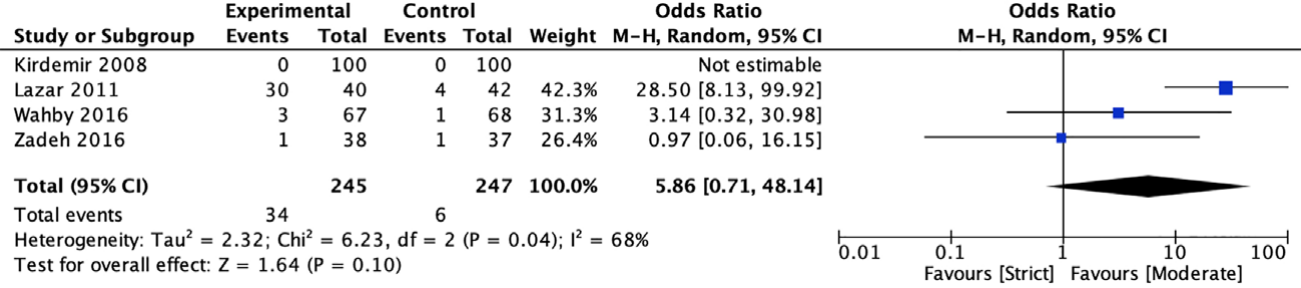
Forest plot for postoperative hypoglycemic episodes between perioperative strict and moderate glycemic control strategy.

Four studies (20, 22, 40, 41) reported the incidence of hypoglycemic episodes between strict and moderate glycemic control strategy. The pooled results suggested there was no significant reduction in the incidence of hypoglycemic episodes [13.88 *vs* 2.43%, OR = 5.86, 95%CI (0.71, 48.14), *P* = 0.10) between strict and moderate glycemic control strategy and there was a significant heterogeneity in the pooled estimates (*I* ^2^ = 68%, *P* = 0.04) (**Figure 6**).

### Moderate *vs* Liberal Glycemic Control Strategy

Only one study (21) reported the incidence of mortality and atrial fibrillation for moderate *versus* liberal glycemic control strategy. There was no death in both groups and the pooled results showed that moderate glycemic control strategy was associated with a significant reduction in atrial fibrillation versus liberal glycemic control strategy [16.67 *vs* 42.03%, OR = 0.28, 95%CI (0.13, 0.60), *P* = 0.001).

### Publication Bias

The number of studies included in each outcome was less than 10, thus, it cannot be attempted to use funnel plot to assess the publication bias. Two studies (22, 41) reported their funding was supported by non-profit research institution. Although one study (21) accepted partial research funding by Eli Lilly, it declared no relevant conflict of interest.

### Quality of Evidence

For the strict *versus* moderate glycemic control strategy, the quality of evidence for the incidence of mortality and stroke is low. The reasons for the downgrade include the risk of bias and imprecision. The quality of evidence for the incidence of atrial fibrillation and sternal wound infection is moderate and its reason for the downgrade is the risk of bias. The quality of evidence for the incidence of hypoglycemic episodes is very low. The reasons for the downgrade include the risk of bias, imprecision, and inconsistency. For the moderate versus liberal glycemic control strategy, the quality of evidence for the incidence of atrial fibrillation is low and its reasons for the downgrade include the risk of bias and imprecision (The detailed information of GRADE is in **Supplementary 3**).

## Discussion

According to the Chinese Heart Survey, the total prevalence of abnormal glucose metabolism in hospitalized patients with coronary heart disease is 76.9% (42). Because of multiple coronary artery lesions, some patients may require cardiac surgery, including coronary artery bypass grafting. In addition, abnormal glucose metabolism also accounts for a large proportion of other heart conditions, such as severe structural heart disease and end-stage heart failure. Considering that hyperglycemia, hypoglycemia, and persistent glycemic fluctuations may increase the mortality rate, incidence of infection, wound healing and complications of various cardio-cerebral events and so on (43–46). Therefore, reasonable perioperative glycemic management strategy is the key to successful operation and good prognosis in patients with diabetes undergoing cardiac surgery.

In this meta-analysis, we analyzed the effect of liberal, moderate and strict glycemic control strategy in diabetic patients undergoing cardiac surgery. Based on the analysis of included studies, moderate quality of evidence indicated that the strict glycemic control strategy was associated with a significant reduction in atrial fibrillation and sternal wound infection, but very low to low-quality of evidence showed that there was no significant reduction in the incidence of mortality, stroke and hypoglycemic episodes. The results of the meta-analysis conducted by Haga et al. (47) in a mixed population of patients with and without diabetes undergoing cardiac surgery showed strict glycemic control can reduce the incidence of ICU mortality and post-surgical atrial fibrillation. We considered the difference in the incidence of mortality was due to the fact that most of the studies we analyzed had mortality within 30 days after surgery, while their examination was performed in the ICU. The results of Sathya’s review (48) are also different with our results. Their analysis indicated that when compared with liberal target (>200 mg/dl), moderate perioperative glycemic target (150–200 mg/dl) was associated with reduction of postoperative mortality and stroke for patients with diabetes, but there were no significant differences between moderate and strict (100–150 mg/dl). We believe this is because their review included not only diabetics, but also some non-diabetics, which led to their patients with lower risk for postoperative outcomes and more likely to achieve glycemic control targets than ours.

Stroke is the main cause of death for patients with diabetes undergoing cardiac surgery, up to 30% (49), and the risk of postoperative mortality may be consistent with stroke for diabetics. The pooled results of our review indicated that strict and moderate glycemic control strategies had no significant difference in the incidence of stroke and mortality. A meta-analysis conducted by Marik et al. (50) focused on the critically ill patients with and without diabetes. Their results were consistent with ours, showing that there was no difference in mortality between intervention group (103–124 mg/dl) and control group (139–171 mg/dl). In addition, their results showed that the incidence of hypoglycemic events was seven times higher in the intervention group than in the control group, which is different from our results that found no difference. Combined with clinical practice, we think this may be due to the low incidence of hypoglycemia in the relevant studies and more researches are needed to further evaluate the risks of hypoglycemic episodes. Although it is still controversial, we speculate that any potential benefits of a strict glycemic control may be offset by the potential deleterious effects of perioperative hypoglycemia, which is more common in the strict glycemic control group (21, 51, 52). Our review highlights the importance of strict glycemic control for reducing the incidence of postoperative atrial fibrillation and wound infection. The results of previous meta-analyses, which included both diabetics and non-diabetics undergoing surgery, were consistent with our findings (47).

However, our review also has some potential limitations. We only searched articles published in Chinese and English. Although international studies were included, it is not clear whether studies in other languages could be selected for our meta-analysis. The small sample size and single center of RCT included in this meta-analysis may limit the generalizability or statistical validity of the results. Moreover, there were differences in the timing of interventions (preoperative or intraoperative), and the length of postoperative mortality follow up was also variable (ranged from in-hospital only to 30-days).

## Conclusions

Our review showed that when compared with moderate target (140–180 mg/dl or 7.8–10.0 mmol/L), maintaining strict glycemic control strategy (<140 mg/dl or 7.8 mmol/L) in patients with diabetes undergoing cardiac surgery was associated with lower risk of atrial fibrillation and sternal wound infection. No benefit was found with liberal glycemic control strategy (>180 mg/dl or 10.0 mmol/L), so it could be a poor glycemic control strategy. While several RCTs have been conducted, more are needed to confirm and extend these results. Although there were several confounders in this study that may weaken the evidence quality of the meta-analysis, we believe the findings from this study provide valuable information with regards to outcomes in patients with diabetes undergoing cardiac surgery.

## Data Availability Statement

Publicly available datasets were analyzed in this study. This data can be found here: doi: 10.1053/j.jvca.2007.09.015; Tight glycemic control in diabetic coronary artery bypass graft patients improves perioperative outcomes and decrease recurrent ischemic events; doi: 10.1097/SLA.0b013e31822c5d78; Effect of perioperative control of blood glucose level on patient’s outcome after anesthesia for cardiac surgery. *Egypt J Anaesth* (2013) 29:71-6; Perioperative glycemic control in diabetic patients undergoing coronary artery bypass graft surgery. *Journal of the Egyptian Society of Cardio-Thoracic Surgery* (2016) 24:143-9; A Study on the Outcomes of Modified Tight Glucose Control for the Management of Glycemic Control in Diabetic Patients Undergoing Cardiac Surgery. *Journal of Pharmacy Research* (2016) 10:764-70.

## Author Contributions

XJ, JJW, and YFM contributed to study concept and design, search of the literatures, data extraction, data analyses, and the drafting and review of the final manuscript. YMM and YC contributed to the conception and design of the analysis, interpreted and analyzed data. YMM, YC, and KC critically reviewed the manuscript, and helped to draft the manuscript. XL, PA, JW, and WM participated in design of the analysis, data interpretation, and review of the manuscript. All authors contributed to the article and approved the submitted version.

## Funding

This study was supported by the Beijing Municipal Science & Technology Commission (Project No. D141107005314004), the Biotechnology Development Center of China (2016YFC1305200), the Scientific and Technological Innovation Program of Sanya (2016YW31), the Program of the Hainan Academician Team Innovation Center supported by Hainan Science and Technology Agency, and the National Science and Technology Major Project (2018ZX09201-013).

## Conflict of Interest

The authors declare that the research was conducted in the absence of any commercial or financial relationships that could be construed as a potential conflict of interest.
