# Supplementary material for: Association Between Perioperative Glycemic Control Strategy and Mortality in Patients With Diabetes Undergoing Cardiac Surgery: A Systematic Review and Meta-Analysis
Source: Front Endocrinol (Lausanne). 2020 Dec 17;11:513073. doi: 10.3389/fendo.2020.513073 (PMC7774648; doi:10.3389/fendo.2020.513073)
Supplement: Supplementary file 1 [file Table_1.docx]

Supplementary Material

# Supplementary 1:Search Strategy

**Medline （n=4207）**

1. "Perioperative Period"[Mesh]
2. "Perioperative Care"[Mesh]
3. "Surgical Procedures, Operative"[Mesh]
4. "perioperative" [Title/Abstract]
5. "peri-operative"[Title/Abstract]
6. "preoperative" [Title/Abstract]
7. "pre-operative"[Title/Abstract]
8. "postoperative" [Title/Abstract]
9. "post-operative"[Title/Abstract]
10. "pre-surgery"[Title/Abstract]
11. "peri-surgery"[Title/Abstract]
12. "post-surgery"[Title/Abstract]
13. "intraoperative" [Title/Abstract]
14. "intra-operative"[Title/Abstract]
15. "surgical" [Title/Abstract]
16. OR/#1-15
17. "Diabetes Mellitus" [MESH]
18. "diabet*" [Title/Abstract]
19. "urophthisis" [Title/Abstract]
20. "glycuresis" [Title/Abstract]
21. "insulin resistance" [Title/Abstract]
22. OR #17-21
23. "Coronary Artery Bypass" [Mesh]
24. "Thoracic surgical procedures" [Mesh]
25. "Thoracic surgical procedures" [Title/Abstract]
26. "Thoracic Surgery" [Title/Abstract]
27. "Aortocoronary Bypass" [Title/Abstract]
28. "Cardiac surgery" [Title/Abstract]
29. "Cardiac surgical procedures" [Title/Abstract]
30. "Cardiovascular Surgical Procedure" [Title/Abstract]
31. "CABG" [Title/Abstract]
32. "coronary surgery" [Title/Abstract]
33. "coronary artery bypass" [Title/Abstract]
34. "heart surgery" [Title/Abstract]
35. "Heart Surgical Procedure" [Title/Abstract]
36. "thoracic surgery" [Title/Abstract]
37. "thoracic surgical procedures" [Title/Abstract]
38. OR #23-37
39. #16 AND #22AND 38

**EBMSAE （n=235）**

1. 'perioperative period'/exp
2. 'perioperative nursing'/exp
3. 'surgery'/exp
4. 'perioperative':ab,ti
5. 'peri-operative':ab,ti
6. 'preoperative':ab,ti
7. 'pre-operative': ab,ti
8. 'postoperative':ab,ti
9. 'post-operative':ab,ti
10. 'pre-surgery':ab,ti
11. 'peri-surgery’:ab,ti
12. 'post-surgery':ab,ti
13. 'intraoperative':ab,ti
14. 'intra-operative':ab,ti
15. 'surgical':ab,ti
16. OR #1-15
17. 'Diabetes Mellitus'/exp
18. 'diabet*':ti ab
19. 'urophthisis':ti ab
20. 'glycuresis':ti ab
21. 'insulin resistance':ti ab
22. OR #17-21
23. 'coronary artery bypass graft'/exp
24. 'Thoracic surgical procedures': ab,ti
25. 'Thoracic Surgery': ab,ti
26. 'Aortocoronary Bypass': ab,ti
27. 'Cardiac surgery': ab,ti
28. 'Cardiac surgical procedures': ab,ti
29. 'Cardiovascular Surgical Procedure': ab,ti
30. 'CABG': ab,ti
31. 'coronary surgery': ab,ti
32. 'coronary artery bypass': ab,ti
33. 'heart surgery': ab,ti
34. 'Heart Surgical Procedure': ab,ti
35. 'thoracic surgery': ab,ti
36. 'thoracic surgical procedures': ab,ti
37. OR #23-36
38. #16 AND #22 AND 37
39. #38/medline
40. #38 NOT #39

**Cochrane library （n=440）**

1. MeSH descriptor: [Surgical Procedures, Operative] explode all trees
2. "perioperative":ti,ab,kw
3. "peri-operative":ti,ab,kw
4. "preoperative":ti,ab,kw
5. "pre-operative":ti,ab,kw
6. "postoperative":ti,ab,kw
7. "post-operative":ti,ab,kw
8. "pre-surgery":ti,ab,kw
9. "peri-surgery":ti,ab,kw
10. "post-surgery":ti,ab,kw
11. "intraoperative":ti,ab,kw
12. "intra-operative":ti,ab,kw
13. "surgical":ti,ab,kw
14. OR/#1-13
15. MeSH descriptor: [Diabetes Mellitus] explode all trees
16. "diabet*":ti,ab,kw
17. "urophthisis":ti,ab,kw
18. "glycuresis":ti,ab,kw
19. "insulin resistance":ti,ab,kw
20. OR#15-19
21. MeSH descriptor: [Coronary Artery Bypass] explode all trees
22. "Thoracic surgical procedures":ti,ab,kw
23. "Thoracic Surgery":ti,ab,kw
24. "Aortocoronary Bypass":ti,ab,kw
25. "Cardiac surgery":ti,ab,kw
26. "Cardiac surgical procedures":ti,ab,kw
27. "Cardiovascular Surgical Procedure":ti,ab,kw
28. "CABG":ti,ab,kw
29. "coronary surgery":ti,ab,kw’
30. "coronary artery bypass":ti,ab,kw
31. "heart surgery":ti,ab,kw
32. "Heart Surgical Procedure":ti,ab,kw
33. "thoracic surgery":ti,ab,kw
34. "thoracic surgical procedures":ti,ab,kw
35. OR#21-34
36. #14 AND #20 AND 35

**Web of Science（n=362）**

1. TOPIC: "perioperative"
2. TOPIC:"peri-operative"
3. TOPIC: "preoperative"
4. TOPIC:"pre-operative"
5. TOPIC: "postoperative"
6. TOPIC:"post-operative"
7. TOPIC:"pre-surgery"
8. TOPIC:"peri-surgery"
9. TOPIC:"post-surgery"
10. TOPIC:"intraoperative"
11. TOPIC:"intra-operative"
12. TOPIC: "surgical"
13. OR/#1-12
14. TOPIC: "Diabetes Mellitus"
15. TOPIC: "diabet*"
16. TOPIC: "urophthisis"
17. TOPIC: "glycuresis"
18. TOPIC: "insulin resistance"
19. OR #14-18
20. TOPIC: "Thoracic surgical procedures"
21. TOPIC: "Thoracic Surgery"
22. TOPIC: "Aortocoronary Bypass"
23. TOPIC: "Cardiac surgery"
24. TOPIC: "Cardiac surgical procedures"
25. TOPIC: "Cardiovascular Surgical Procedure"
26. TOPIC: "CABG"
27. TOPIC: "coronary surgery"
28. TOPIC: "coronary artery bypass"
29. TOPIC: "heart surgery"
30. TOPIC: "Heart Surgical Procedure"
31. TOPIC: "thoracic surgery"
32. TOPIC: "thoracic surgical procedures"
33. OR#20-32
34. #13 AND #19 AND #33

**Chinese Database**

**CNKI（n=149）**

1. 心脏手术[主题]
2. 心脏外科[主题]
3. 冠脉搭桥术[主题]
4. 冠状动脉旁路移植术[主题]
5. 心脏瓣膜置换术[主题]
6. 心肌灌注[主题]
7. 心肌再灌注[主题]
8. 搭桥手术[主题]
9. OR #1-8
10. 糖尿病[主题]
11. 术前[主题]
12. 术中[主题]
13. 术后[主题]
14. 围手术期[主题]
15. OR #11-14
16. #9 AND #10 AND #15

**万方（n=618）**

1. 心脏手术[主题]
2. 心脏外科[主题]
3. 冠脉搭桥术[主题]
4. 冠状动脉旁路移植术[主题]
5. 心脏瓣膜置换术[主题]
6. 心肌灌注[主题]
7. 心肌再灌注[主题]
8. 搭桥手术[主题]
9. OR #1-8
10. 糖尿病[主题]
11. 术前[主题]
12. 术中[主题]
13. 术后[主题]
14. 围手术期[主题]
15. OR #11-14
16. #9 AND #10 AND #15

**CBM（n=303）**

1. 心脏手术[常用字段]
2. 心脏外科[常用字段]
3. 冠脉搭桥术[常用字段]
4. 冠状动脉旁路移植术[常用字段]
5. 心脏瓣膜置换术[常用字段]
6. 心肌灌注[常用字段]
7. 心肌再灌注[常用字段]
8. 搭桥手术[常用字段]
9. OR #1-8
10. 糖尿病[不加权:扩展]
11. 糖尿病[常用字段]
12. OR #10-11
13. 手术期间[不加权:扩展]
14. 手术期间[常用字段]
15. 术前[常用字段]
16. 术中[常用字段]
17. 术后[常用字段]
18. 围手术期[常用字段]
19. OR #13-18
20. #9 AND #12 AND #19

# Supplementary 2:References to studies excluded from this review

**Comparison of non-different glycemic level control strategy: *n*=9**

1. Emam I A , Allan A , Eskander K , et al. Our experience of controlling diabetes in the peri-operative period of patients who underwent cardiac surgery. Diabetes research and clinical practice, 2010, 88(3):242-246.
2. Barcellos C D S D , Wender O C B , Azambuja P C D . Clinical and hemodynamic outcome following coronary artery bypass surgery in diabetic patients using glucose-insulin-potassium (GIK) solution: a randomized clinical trial. Rev Bras Cir Cardiovasc, 2007, 22(3):275-284.
3. Li J Y, Sun S, Wu S J. Continuous insulin infusion improves postoperative glucose control in patients with diabetes mellitus undergoing coronary artery bypass surgery.. Tex Heart Inst J, 2006, 33(4):445-451.
4. Ouattara A , Lecomte P , Manach Y L , et al. Poor Intraoperative Blood Glucose Control Is Associated with a Worsened Hospital Outcome after Cardiac Surgery in Diabetic Patients | Anesthesiology | ASA Publications. Anesthesiology, 2005, 103(4):687.
5. Abelev Z, Seth A, Patel R, et al. Continuous insulin infusion is associated with a reduced post-surgical length of stay, but not with the complication rate, in patients with diabetes mellitus undergoing coronary artery bypass graft. J Endocrinol Invest. 2011 Nov;34(10):770-4.
6. Casper H Jørgensen, Gislason GH , Andersson C, et al. Effects of oral glucose-lowering drugs on long term outcomes in patients with diabetes mellitus following myocardial infarction not treated with emergent percutaneous coronary intervention - a retrospective nationwide cohort study. Cardiovascular Diabetology, 2010, 9(1):54.
7. Silinskie KM, Kirshner R, Hite MS. Converting Continuous Insulin Infusion to Subcutaneous Insulin Glargine After Cardiac Surgery Using Percentage-Based Versus Weight-Based Dosing: A Pilot Trial. Annals of Pharmacotherapy, 2013, 47(1):20-8.
8. Omar AS, Salama A, Allam M, et al. Association of time in blood glucose range with outcomes following cardiac surgery. BMC Anesthesiol. 2015, 26;15:14.
9. Stephens JW, Krause AH, Peterson CA, et al. The Effect of Glucose Priming Solutions in Diabetic Patients Undergoing Coronary Artery Bypass Grafting. Annals of Thoracic Surgery, 1988, 45(5):544-547.

**Containing Non-diabetic Patients: *n*=11**

1. Chan RP, Galas FR, Hajjar LA, et al. Intensive perioperative glucose control does not improve outcomes of patients submitted to open-heart surgery: a randomized controlled trial. Clinics, 2009, 64(1):51-60.
2. Desai SP, Henry LL, Holmes SD, et al. Strict versus liberal target range for perioperative glucose in patients undergoing coronary artery bypass grafting: A prospective randomized controlled trial. J Thorac Cardiovasc Surg, 2012, 143(2):318-325.
3. Gandhi GY, Nuttall GA, Abel MD, et al. Intensive Intraoperative Insulin Therapy versus Conventional Glucose Management during Cardiac Surgery: A Randomized Trial. Annals of Internal Medicine, 2007, 146(4):233-43.
4. Corpus RA, George PB, House JA, et al. Optimal glycemic control is associated with a lower rate of target vessel revascularization in treated type II diabetic patients undergoing elective percutaneous coronary intervention. Journal of the American College of Cardiology, 2004, 43(1):8-14.
5. Bhamidipati CM, Lapar DJ, Stukenborg GJ, et al. Superiority of moderate control of hyperglycemia to tight control in patients undergoing coronary artery bypass grafting. Journal of Thoracic & Cardiovascular Surgery, 2011, 141(2):543-551.
6. Reyes-Umpierrez D, Davis G, Cardona S, et al. Inflammation and Oxidative Stress in Cardiac Surgery Patients Treated to Intensive Versus Conservative Glucose Targets. Journal of clinical endocrinology and metabolism, 2017, 102(1): 309-315.
7. Umpierrez G, Cardona S, Pasquel F, et al. Randomized Controlled Trial of Intensive Versus Conservative Glucose Control in Patients Undergoing Coronary Artery Bypass Graft Surgery: GLUCO-CABG Trial. Diabetes Care, 2015, 38(9):1665-72.
8. Smith CE, Styn N, Kalhan S, et al. Intraoperative glucose control in diabetic and nondiabetic patients during cardiac surgery. J Cardiothorac Vasc Anesth, 2005, 19(2):201-208.
9. Zimmerman CR, Mlynarek ME, Jordan JA, et al. An Insulin Infusion Protocol in Critically Ill Cardiothoracic Surgery Patients. Annals of Pharmacotherapy, 2004, 38(7):1123-1129.
10. Goldberg PA, Sakharova OV, Barrett PW, et al. Improving glycemic control in the cardiothoracic intensive care unit: clinical experience in two hospital settings.. Journal of Cardiothoracic & Vascular Anesthesia, 2004, 18(6):690-697.
11. Gong WH, Shi KH, Zhang F, et al. Effect of intensive insulin therapy on extracorporeal circulation coronary artery bypass grafting in elderly non-diabetic patients. Journal of Chinese Physician, 2011, 13(5):638-644. [Article in Chinese]

**Missing concerned outcomes: *n*=1**

1. Kuskonmaz SM, Kurtipek Ö, Aydın ME, et al. A retrospective analysis of blood gases with two different insulin infusion protocols in patients undergoing cardiovascular surgery. Nigerian Journal of Clinical Practice, 2016, 19(6):742-6.

# Supplementary 3: Quality assessment using the GRADE approach

| **Outcomes** | **No of participants (studies)** | **Quality of the evidence (GRADE)** | **Relative effect (95% CI)** | **Anticipated absolute effects** | |
| --- | --- | --- | --- | --- | --- |
|  |  |  |  | **Risk with control group** | **Risk difference with observation group** |
| **Strict vs Moderate glycemic level control strategy** | | | | | |
| Mortality | 492(4 RCTs) | ⨁⨁◯◯  LOW^1,2^ | OR 0.57  (0.20 to 1.66) | 36 per 1,000 | **15 fewer per 1,000**(29 fewer to 23 more) |
| Stroke | 375(3 RCTs) | ⨁⨁◯◯  LOW^1,2^ | OR 0.70  (0.14 to 3.62) | 16 per 1,000 | **5 fewer per 1,000**(14 fewer to 40 more) |
| Atrial fibrillation | 592(5 RCTs) | ⨁⨁⨁◯  MODERATE^1^ | OR 0.48  (0.32 to 0.72) | 286 per 1,000 | **125 fewer per 1,000**(172 fewer to 62 fewer) |
| Sternal Wound infection | 492(4 RCTs) | ⨁⨁⨁◯  MODERATE^1^ | OR 0.28  (0.14 to 0.54) | 166 per 1,000 | **113 fewer per 1,000**(139 fewer to 69 fewer) |
| Hypoglycemic episodes | 492(4 RCTs) | ⨁◯◯◯  VERY LOW^1,2,3^ | OR 5.86  (0.71 to 48.14) | 24 per 1,000 | **103 more per 1,000**(7 fewer to 521 more) |
| **Moderate vs liberal glycemic level control strategy** | | | | | |
| Atrial fibrillation | 141 (1 RCT) | ⨁⨁◯◯  LOW^4,5^ | OR 0.28  (0.13 to 0.60) | 420 per 1,000 | **252 fewer per 1,000**(334 fewer to 117 fewer) |
| ***The risk in the intervention group** (and its 95% confidence interval) is based on the assumed risk in the comparison group and the **relative effect** of the intervention (and its 95% CI).  **CI:** Confidence interval; **OR:** Odds ratio | | | | | |
| **GRADE Working Group grades of evidence** **High quality:** We are very confident that the true effect lies close to that of the estimate of the effect **Moderate quality:** We are moderately confident in the effect estimate: The true effect is likely to be close to the estimate of the effect, but there is a possibility that it is substantially different **Low quality:** Our confidence in the effect estimate is limited: The true effect may be substantially different from the estimate of the effect **Very low quality:** We have very little confidence in the effect estimate: The true effect is likely to be substantially different from the estimate of effect | | | | | |
| **Notes:**   1. Downgraded one level for risk of bias: high risk of random sequence in a study; high risk of blinding of participants and personnel in all studies. 2. Downgraded one level for imprecision: 95% CI includes both no effect and appreciable benefit. 3. Downgraded one level for inconsistency: the value of square I above 50%. 4. Downgraded one level for risk of bias: high risk of random sequence in the study.   Downgraded one level for imprecision: The sample size is less than the optimal information sample size. | | | | | |
